# *PRPH2*-Related Retinal Diseases: Broadening the Clinical Spectrum and Describing a New Mutation

**DOI:** 10.3390/genes11070773

**Published:** 2020-07-09

**Authors:** Rosa M. Coco-Martin, Hortensia T. Sanchez-Tocino, Carmen Desco, Ricardo Usategui-Martín, Juan J. Tellería

**Affiliations:** 1 Instituto Universitario de Oftalmobiologia Aplicada, Universidad de Valladolid, 47011 Valladolid, Spain; rusateguim@ioba.med.uva.es (R.U.-M.); juanjose.telleria@uva.es (J.J.T.); 2 Red Temática de Investigación Cooperativa en Salud de Oftalmologia (Oftared), Instituto de Salud Carlos III, 28029 Madrid, Spain; 3 Department of Ophthalmology, Hospital Universitario Rio Hortega, 47012 Valladolid, Spain; tensi_sanchez@yahoo.es; 4 Fisabio Oftalmologia Medica, 46035 Valencia, Spain; carmen.desco@uv.es

**Keywords:** *PRPH2*, *ABCA4*, AVMD, pattern dystrophy simulating FF, extensive chorioretinal atrophy, CACD, blended phenotypes, inherited retinal diseases

## Abstract

Over 175 pathogenic mutations in the Peripherin-2 (*PRPH2)* gene are linked to various retinal diseases. We report the phenotype and genotype of eight families (24 patients) with retinal diseases associated with seven distinct *PRPH2* gene mutations. We identified a new mutation, c.824_828+3delinsCATTTGGGCTCCTCATTTGG, in a patient with adult-onset vitelliform macular dystrophy (AVMD). One family with the p.Arg46Ter mutation presented with the already described AVMD phenotype, but another family presented with the same mutation and two heterozygous pathogenic mutations (p.Leu2027Phe and p.Gly1977Ser) in the ATP Binding Cassette Subfamily A Member 4 (*ABCA4*) gene that cause extensive chorioretinal atrophy (ECA), which could be a blended phenotype. The p.Lys154del *PRPH2* gene mutation associated with the p.Arg2030Glu mutation in the *ABCA4* gene was found in a patient with multifocal pattern dystrophy simulating fundus flavimaculatus (PDsFF), for whom we considered *ABCA4* as a possible modifying gene. The mutation p.Gly167Ser was already known to cause pattern dystrophy, but we also found ECA, PDsFF, and autosomal-dominant retinitis pigmentosa (ADRP) as possible phenotypes. Finally, we identified the mutation p.Arg195Leu in a large family with common ancestry, which previously was described to cause central areolar choroidal dystrophy (CACD), but we also found ADRP and observed that it caused ECA more frequently than CACD in this family.

## 1. Introduction

Mutations in the Peripherin-2 (*PRPH2*) gene (OMIM: 179605) are among the most frequently found in inherited retinal diseases (IRD) [1], with an even higher percentage among diseases primarily involving the central retina [2]. The mutations may differentially affect the gene’s protein product role as a structural component or as a functional protein that is key for organizing membrane domains for cellular signaling. These roles may be different in the rods and cones, thus contributing to the phenotypic heterogeneity that characterizes this group of diseases [3].

Over 175 pathogenic mutations in the *PRPH2* gene are linked to numerous human retinal diseases (summarized at http://www.retina-international.org/files/sci-news//rdsmut.htm) [4], which generally have an autosomal-dominant inheritance pattern. Their linked phenotypes show significant variability in age at onset, severity, and a range of clinical features including those limited to the macula, such as adult-onset vitelliform macular dystrophy (AVMD, MIM 608161), butterfly patterned dystrophy (PD, MIM 169150), or central areolar choroidal dystrophy (CACD, MIM 613105), and those with more widespread disorders, such as retinitis pigmentosa 7 (RP, MIM 608133) or retinitis punctata albescens (MIM 136880) or digenic RP caused by heterozygous mutations in Retinal Outer Segment Membrane Protein 1 (*ROM1*) and *PRPH2* genes in conjunction. Homozygous *PRPH2* mutations cause Leber’s congenital amaurosis 18 (MIM 608133). Finally, clinical features also may look similar to the flecked retina associated with mutations of the ATP Binding Cassette Subfamily A Member 4 gene (*ABCA4*), which in this case is called multifocal patterned dystrophy simulating fundus flavimaculatus (PDsFF) [5]. Moreover, great variability has been reported even in single-point mutations among members of the same family who may present with very distinct phenotypes [6,7].

Mutations in the *PRPH2* gene are associated with marked phenotypic heterogeneity and show relatively limited genotype–phenotype correlation [8]. This is because there is transition from one clinical classification to another as patients grow older and is also due to the inter- and intra-familial phenotypic variability, even among all patients carrying the same mutant allele. Currently, no known treatment has been developed for these diseases and the presence of other genetic modifiers (*ROM1*, *ABCA4*, etc.) makes gene therapy design challenging. Therefore, studying these conditions is important to identify new ways to improve vision in these patients. Knowing the mutations that patients carry also is important for genetic counseling. In this study, we present a detailed clinical characterization of 24 patients to broaden the spectrum of molecularly confirmed macular dystrophy due to *PRPH2* mutations by disclosing new clinical presentations of known mutations, one new mutation, and one possible blended phenotype.

## 2. Materials and Methods 

### 2.1. Ethics Statement

This observational cross-sectional study was conducted in accordance with the 1964 Declaration of Helsinki and its subsequent amendments. All subjects signed an informed consent form, including consent to publish photographs, under protocol code number IOBA-2020-D, approved by the Instituto Universitario de Oftalmobiologia Aplicada (IOBA) Research Commission. The study was performed at the IOBA Retina Unit, University of Valladolid, Spain.

### 2.2. Clinical Characterization of the Study Subjects

The current study included only Spanish Caucasian patients with a demonstrated mutation in the *PRPH2* gene from a database of 579 patients. Patients underwent a routine ocular examination at IOBA. Patient demographic data, age at symptom onset, best-corrected visual acuity (BCVA), fundus appearance, and the results of autofluorescence (AF), spectral-domain optical coherence tomography (SD-OCT), and sometimes fluorescein angiography (FA) were recorded. A detailed family history was obtained from the probands and/or their relatives; first-degree relatives were examined when possible. Automated static perimetry to examine the visual fields (VF) was performed using the Humphrey Field Analyzer (Carl Zeiss Meditec, Dublin, CA, USA). Full-field electroretinogram (ffERG) recordings were assessed using the computerized Optoelectronic Stimulator Vision Monitor MonPack 120 (Metrovision, Pérenchies, France) according to the International Society for Clinical Electrophysiology of Vision protocols [9].

Clinical diagnoses were based on structural and functional eye examinations. AVMD was diagnosed based on the presence of elevated yellow or pigmented deposits between the neurosensory retina and retinal pigment epithelium (RPE) at the foveal or parafoveal region in at least one eye and normal electrophysiology testing. PDsFF was diagnosed in the presence of multiple yellowish, irregular flecks scattered around the posterior pole and mid-periphery, simulating what is observed in FF disease, with a normal ffERG that remains asymptomatic until adulthood. CACD was diagnosed based on the presence of atrophic changes restricted to the macular area that started in middle age. Patients with extensive chorioretinal atrophy (ECA) presented with large atrophic retinal areas involving the macula and fundus mid-periphery, a large central scotoma in the VF, and both subnormal photopic and scotopic ffERGs. Finally, patients presenting with these later features, but associated with night blindness, concentric VF restriction, and an abolished scotopic ffERG with a subnormal, but still present, response in the photopic ffERG were diagnosed with ADRP.

### 2.3. Genetic Analysis

Peripheral blood samples were collected from the affected patients and available unaffected relatives for DNA extraction. For families 1, 2, 5, 6, and 7, the *PRPH2* gene was analyzed by direct sequencing. Forward and reverse direct sequencing was done using Big Dye v3.1 chemistry (Applied Biosystems, Foster City, CA, USA). Capillary electrophoresis was performed on the ABI PRISM 3130xl Genetic Analyzer (Thermo Fisher Scientific, Waltham, MA, USA). The resulting data were analyzed using Sequencing Analysis v5.2 and SeqScape v2.5 software (Applied Biosystems, Foster City, CA, USA). 

Regarding families 3, 4, and 8, DNA was sequenced with an Illumina HiSeq next generation sequencing (NGS) platform (Illumina, San Diego, CA, USA) using a specific IRD enrichment panel based on exon capture technology. Exome enrichment was carried out with the Nextera Exome Kit, previously known as the TruSeq Rapid Exome Library Prep Kit. The IRD panel captured 346 hereditary eye disease genes, including 346 genes and 66 non-coding genomic regions of interest related to more than 50 IRDs including the ones diagnosed in the probands. Several bioinformatic filtering steps were carried out to rule out neutral variants and prioritize possible pathogenic variants. This prioritization was based on genetic and population criteria and complemented by exhaustive bibliographic studies and databases. The mean coverage in the presented cases was 577.2x, and in all three cases, 99.8% of the regions of interest had a read depth of 15x or greater. Stratified disease-associated variants were confirmed by Sanger sequencing. In this study, only those variants that were identified as pathogenic (frameshift, premature stop codon, splice site variants affecting canonical sites, and reported variants known to cause retinal diseases in ClinVar (https://www.ncbi.nlm.nih.gov/clinvar/) or the Human Gene Mutation Database (https://go.qiagen.com/HGMD) or whose frequency in the control population was less than 0.5% and complied with the pathogenicity predictions following established bioinformatic algorithms such as PolyPhen-2 [10], SIFT [11], and MutationTaster [12] were reported. All the variants reported in this publication had been previously described as pathogenic or likely pathogenic mutations with only one exception. Cases in which two mutated alleles on the same gene were identified would have needed a segregation analysis to identify if they had been inherited separately from the parents, but in most cases of this series, parents were not available for the study. 

## 3. Results

**Figure 1:**
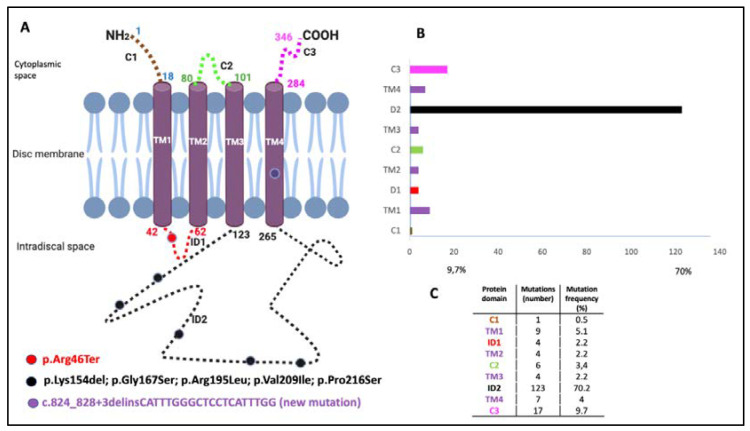
Location of identified mutations on the Peripherin-2 (PRPH2) protein and the frequency and location of all reported *PRPH2* gene mutations. The PRPH2 protein scheme shows the location of the identified mutations. (**A**) The PRPH2 peptide chain shows extradiscal (C1, C2, and C3), transmembrane (TM1, TM2, TM3, and TM4), and intradiscal space locations (ID1 and ID2). The numbering indicates the amino acid positions at the boundaries of the domains. The mutations identified in the patients are indicated by circles (i.e., red, p.Arg46Ter; black, p.Lys154del, p.Gly167Ser, p.Arg195Leu, p.Val209Ile, and p.Pro216Ser; and purple for the novel mutation c.824_828+3delinsCATTTGGGCTCCTCATTTGG). (**B**) Representation of the location (**C**) and the frequency and number of all reported *PRPH2* mutations are based on the Human Gene Mutation Database (HGMD 2020.1).

Twenty-four patients from eight families were included with seven distinct *PRPH2* genetic mutations. The phenotype characterization of patients is summarized in Table 1 and Table 2. Genotype data, type of mutation, location in the protein domains, and clinical diagnoses are presented in Table 3. The location of the identified mutations in a scheme of the PRPH2 protein is shown in Figure 1, together with the frequency and location of all the mutations described to date in the *PRPH2* gene.

**Figure 2:**
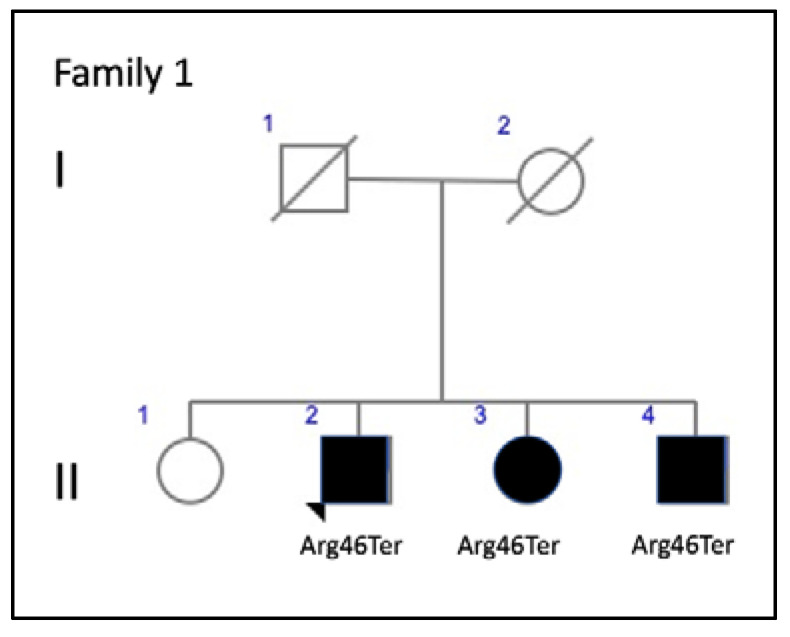
Family 1 pedigree.

**Figure 3:**
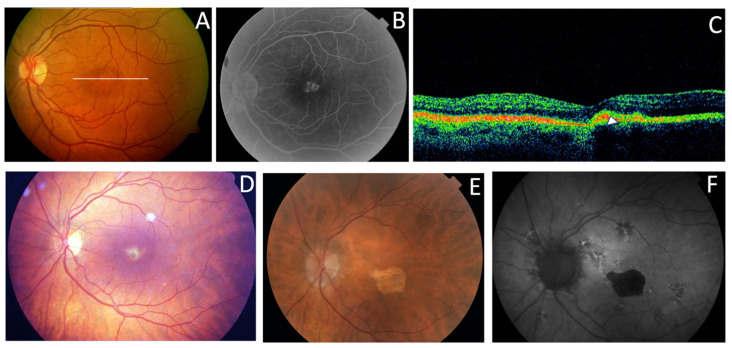
The clinical features of AVMD from families 1 (p.Arg46Ter) and 6 (p.Val209Ile). (**A**) The fundus appearance with the typical small yellow foveal deposits, (**B**) fluorescein angiography (FA) shows the hyperfluorescence produced by a window defect, and (**C**) spectral-domain optical coherence tomography (SD-OCT) shows the subfoveal hyper-reflective deposit (white arrow) from patient F1-II-2. (**D**) The ocular fundus appearance from a patient aged 56 years (F6-I-5) with a pigmented foveal dot surrounded by a yellow halo. (**E**,**F**) The fundus and autofluorescence after 14 years show macular atrophy.

Three affected members from family 1 were siblings with AVMD, aged 56, 51, and 46 years (Figure 2). All three had subretinal foveal and/or parafoveal yellowish deposits either in the fundus or on AF and SD-OCT images (Figure 3A–C). The changes were bilateral and symmetric. All patients reported mild metamorphopsia and the ffERGs were normal. 

**Figure 4:**
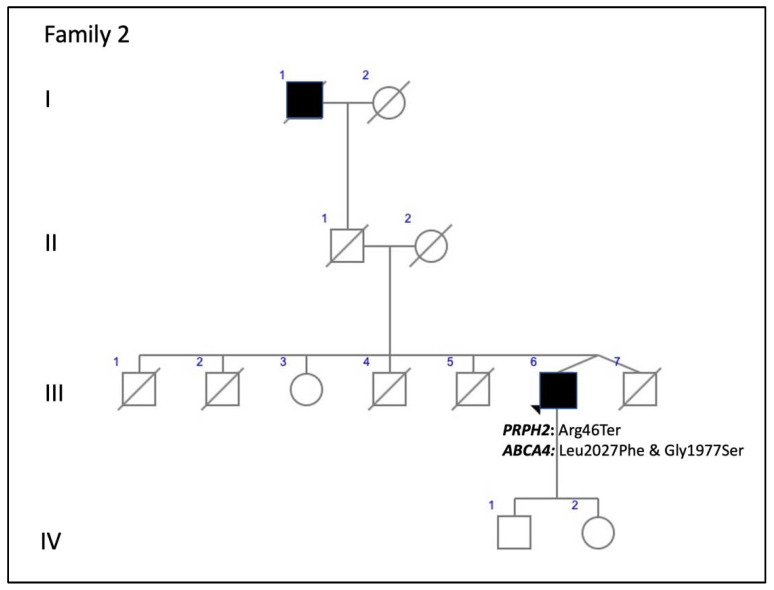
The family 2 pedigree indicates apparent incomplete penetrance.

**Figure 5:**
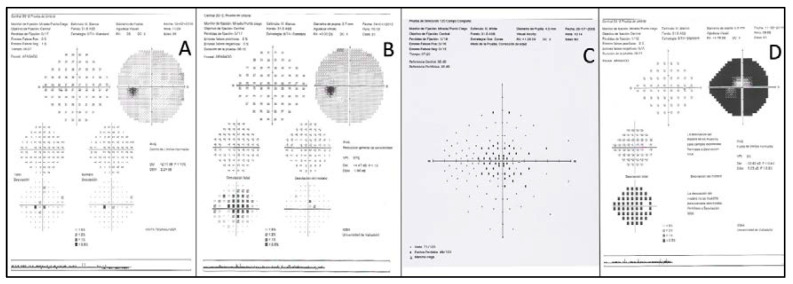
Examples of visual fields (VFs). (**A**) Small paracentral scotoma from F3-V-2 with PDsFF. (**B**) Central scotoma from F5a-III-2 with CACD. (**C**) A large central scotoma from F2-III-6 with ECA. (**D**) Concentric VF restriction from F7-III-1 with ADRP.

**Figure 6:**
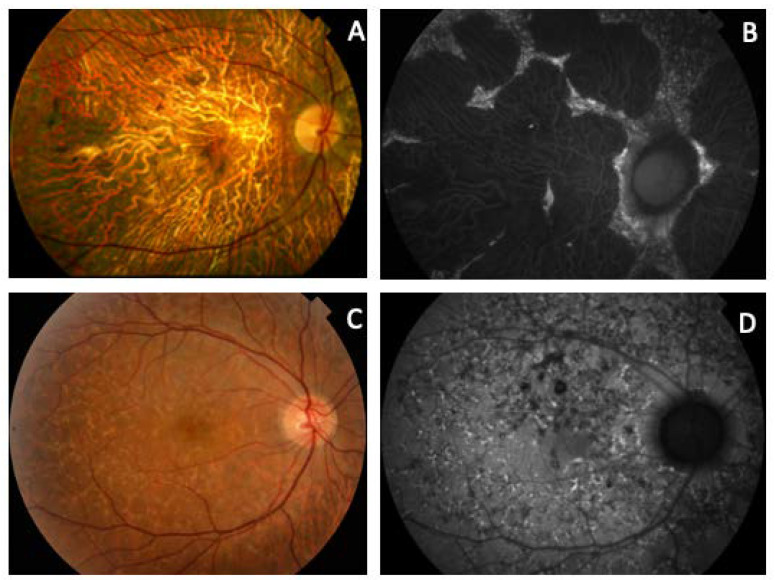
Clinical images from patients with mutations in the *PRPH2* and *ABCA4* genes. (**A**) Fundus and (**B**) autofluorescence of a case from F2-III-6 (*PRPH2*: p.Arg46Ter and *ABCA4*: p.Leu2027Phe and p.Gly1977Ser) with the typical appearance of ECA and foveal preservation that explains the good visual acuity. (**C**) The ocular fundus and (**D**) AF of an F3-V-2 member (*PRPH2*: p.Lys154del and *ABCA4*: p.Arg2030Gln), which resembles FF.

**Figure 7:**
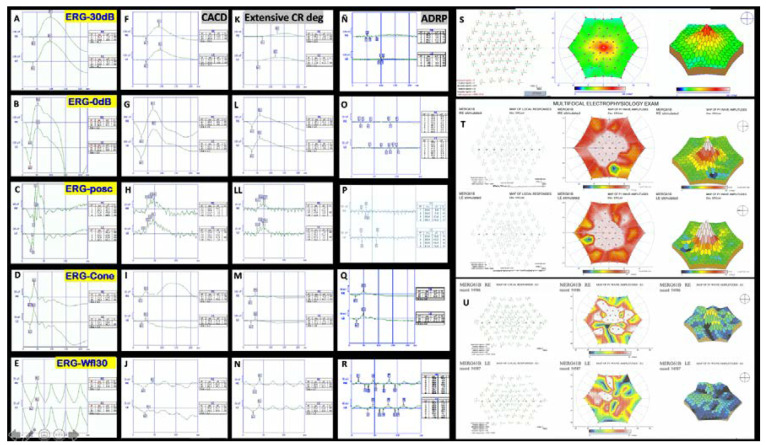
Examples of ERGs. On the left-hand side is a normal ffERG from F8-II-2 (**A**–**E**), and next to it the ffERG shows the decreased photopic and scotopic amplitude in patient F5c-IV-4 with CACD (**F**–**J**). Amplitudes of the *b*-wave are even lower in a case from F2-III-6 with extensive chorioretinal degeneration (**K,L,LL,M,N**). Finally, the ffERG of a case from F7-III-1 with autosomal dominant retinitis pigmentosa (ADRP) shows a flat scotopic and photopic ffERG (**Ñ,O,P,Q,R**). On the right-hand side, the image at the top shows a normal multifocal ERG above (**S**), the ERG from the F3-V-2 patient showing some areas of decreased P1 wave amplitude is in the middle (**T**), and a case from F2-III-6 with ECA showing generalized decrease of the P1 wave amplitude is at the bottom (**U**).

Family 2 included a 52-year-old man with a positive family history (Figure 4). He presented with ECA that produced a large central scotoma (Figure 5), but a well-preserved foveal area on AF throughout life, which explained the good BCVA (Figure 6A,B). Both the scotopic and photopic ffERGs were subnormal (Figure 7). He also had mutations in the *ABCA4* gene (Table 3).

**Figure 8:**
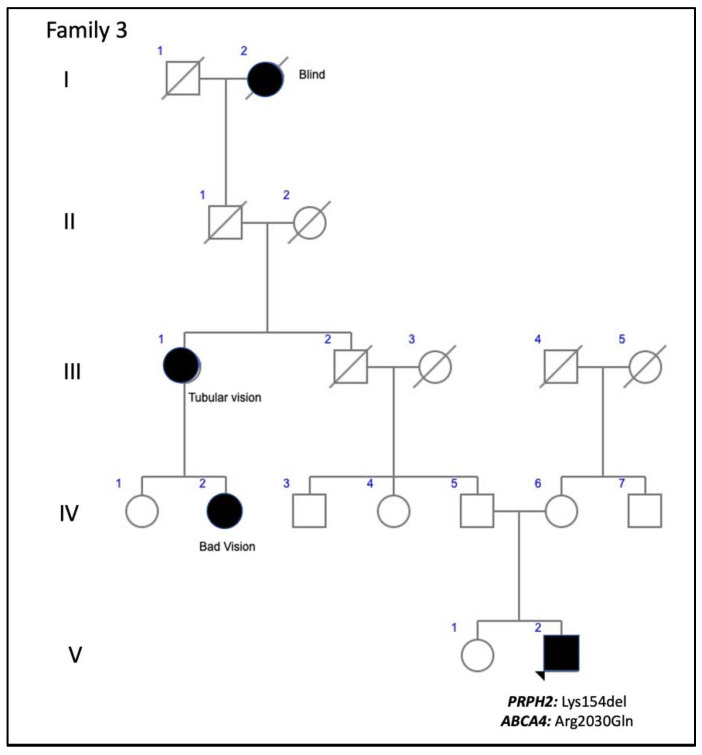
The family 3 pedigree shows apparent non-penetrance.

Family 3 included a 37-year-old man who presented to our clinic for a second opinion to confirm a diagnosis of FF. His paternal great grandmother was blind and had tubular vision, and his paternal great aunt and her two daughters had poor vision. Thus, the pedigree shows apparent non-penetrant inheritance (Figure 8). The patient’s BCVA was 20/16 in the RE and 20/20 in his LE. The VF showed only a mild decrease in sensitivity in the RE. Yellow flecks were seen in the posterior pole and mid-periphery bilaterally. SD-OCT showed an irregularity or mild disruption of the outer nuclear layer and the ellipsoid zone was limited to the foveal region. FA showed a dark choroid and a combination of hypo- and hyper-fluorescent flecks with some areas of extrafoveal incipient retinal atrophy (Figure 6C,D). The scotopic ffERG showed a mild decrease in the *b*-wave amplitude; the photopic ffERG and the multifocal ERG were normal. He also presented with a mutation in allele 1 of the *ABCA4* gene.

**Figure 9:**
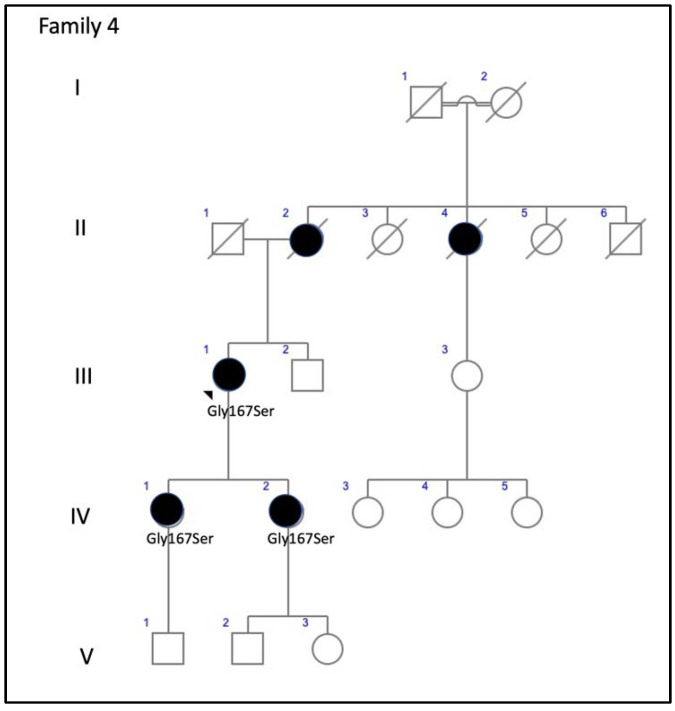
The family 4 pedigree shows a dominant inheritance pattern.

**Figure 10:**
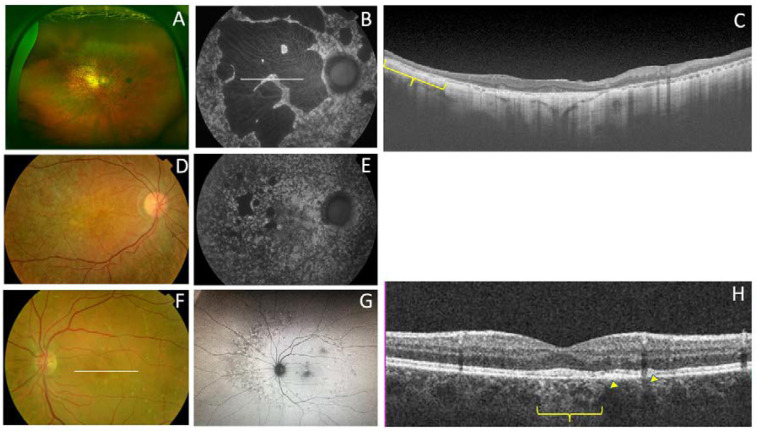
The clinical features of family 4 (p.Gly167Ser). (**A**) The fundus appearance of a case from F4-III-1 shows large confluent areas of chorioretinal atrophy and pigment clumps in mid-periphery, (**B**) AF images with a large area of hypoautofluorescence and speckled points of hypo-AF in the posterior pole and mid-periphery, and (**C**) SD-OCT shows areas of outer retinal atrophy (yellow brace). (**D**) The fundus images obtained from F4-IV-1 show whitish stippling over the posterior pole and (**E**) speckled points of hypo- and hyper-AF in the posterior pole and mid-periphery are seen in her AF. (**F**) The fundus features from F4-IV-2 show yellow triradiate flecks in the posterior pole, (**G**) AF images show scarce speckled hyper-AF and hypo-AF at the posterior pole and mid-periphery, and (**H**) SD-OCT images show disruption of the ellipsoid and interdigitation zones of the juxtafoveal region (yellow arrows) and foveal hypertransmissibility to the choroid (yellow brace).

Family 4 was comprised of a mother and two daughters, aged 78, 53, and 51 years, respectively. The mother reported that her mother and maternal aunt lost vision from the seventh decade of life (Figure 9). The patient F4-III-1 had been diagnosed with macular atrophy at age 68 years after cataract surgery, but achieved 20/25 BCVA bilaterally. The ophthalmologic examination showed small yellowish flecks in the posterior pole with large plaques of outer retinal atrophy that extended to the entire mid-peripheral retina. These changes were bilateral, symmetrical, and much more evident in AF. The atrophic plaques progressed slowly over 10 years; the patient currently has a BCVA of 20/100 in the right eye (RE) and 20/25 in the left eye (LE). Her daughters were asymptomatic and presented with BCVAs of 20/20, but had yellow flecks in the posterior pole that were more numerous in F4-IV-1, who also had small areas of eccentric macular atrophy more evident on AF (Figure 10). The ffERG and VFs were normal in the F4-IV-2 daughter and we diagnosed PDsFF. F4-IV-1 had subnormal photopic and scotopic ffERGs and we diagnosed ECA, but F4-III-1 had an abolished scotopic ffERG with subnormal photopic ffERG and reported night blindness, and therefore we diagnosed ADRP (Figure 7).

**Figure 11:**
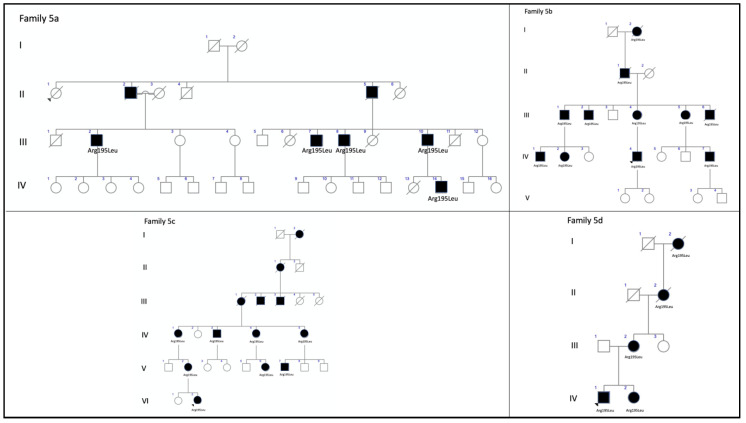
The pedigrees from the four branches of family 5 exhibit a dominant inheritance pattern. The family tree is based on clinical data shown here. The known affected individuals in each family are indicated by a black symbol.

**Figure 12:**
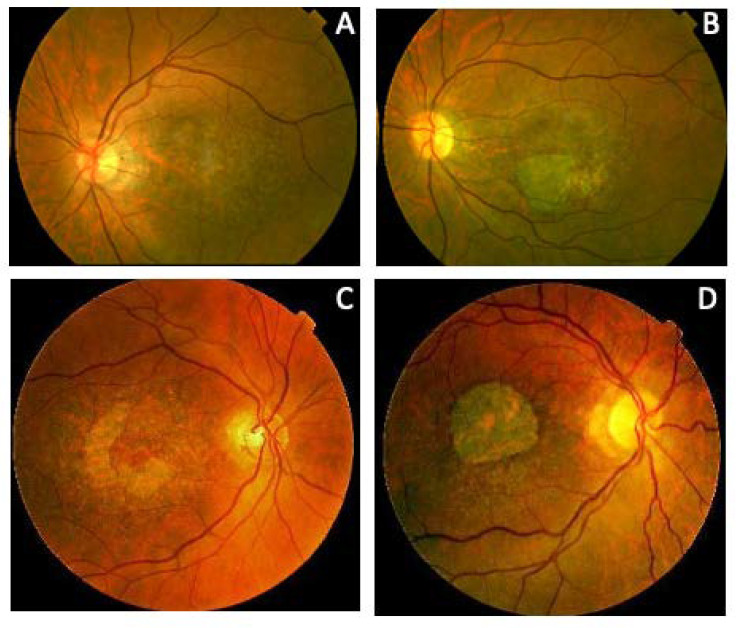
Family 5a (p.Arg195Leu) presents with CACD. (**A**) F5a-III-7 had whitish stippling only in the macular area. (**B**) F5a-III-10 had incipient macular atrophy as well. (**C**) F5a-III-2 had typical foveal preservation. (**D**) F5a-III-6 had total macular atrophy.

**Figure 13:**
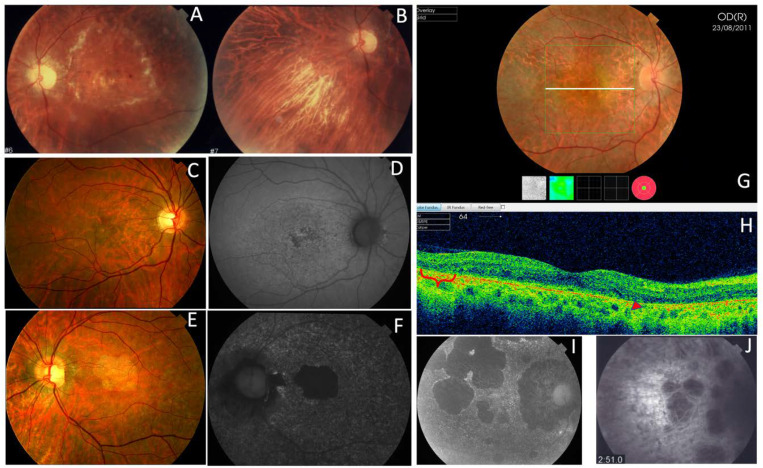
The clinical appearance of family 5b (p.Arg195Leu). F5b-III-4 had (**A**) a large area of atrophy in the posterior pole and (**B**) extensive atrophy in the mid-periphery. The least affected patient was F5b-IV-2 with mild macular changes in the (**C**) fundus and (**D**) AF. F5b-IV-1 had clear macular atrophy in (**E**) the fundus and (**F**) AF. (**G**) F5b-IV-4 had atrophy in the posterior pole and mid-periphery, outer retinal atrophy (red brace and red arrow) was much more evident in the (**H**) SD-OCT and (**I**) autofluorescence images, and (**J**) extension of atrophy to the mid-periphery was evident in the fluorescein angiogram.

**Figure 14:**
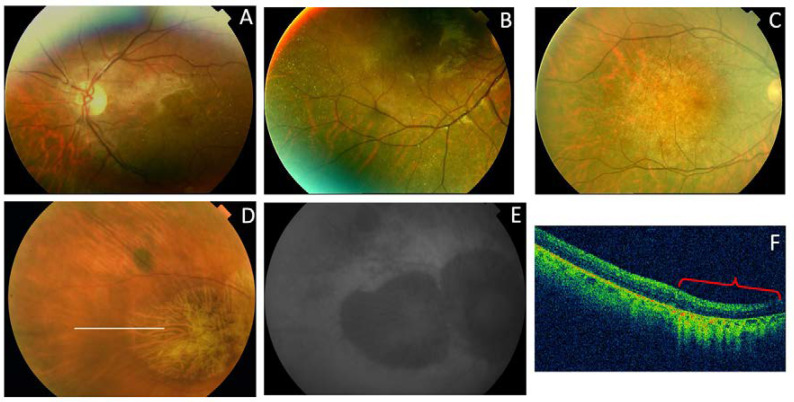
The clinical appearance of family 5c (p.Arg195Leu). (**A**) The posterior pole and (**B**) mid-periphery from F5c-VI-2 had very small whitish dots. (**C**) F5c-V-2 had a more advanced appearance. (**D**) F5c-IV-4 had macular atrophy consistent with the diagnosis of CACD, with evident atrophy in the (**E**) AF and (**F**) SC-OCT (red brace) images.

We also present four different branches of family 5 (Figure 11). Members of this large family had a common ancestor in a small valley in the northern Spanish region of Cantabria. The first branch (family 5a) included three siblings aged 59, 58, and 42 years and their 53-year-old paternal cousin who all presented with CACD (Figure 12). The second branch (family 5b) was comprised of a man, his mother, and two maternal male (F5b-IV-1) and female (F5b-IV-2) cousins. They all presented with ECA that was more aggressive the younger the age of symptom onset and the older the age of each patient. The exception was a 31-year-old female cousin who presented with clinical features of CACD (Figure 13) and a central scotoma (Figure 5), but still had well-preserved vision. The third branch (family 5c) included an 18-year-old woman, her mother, and the mother’s maternal great aunt (F5c-IV-4). The 18-year-old and her mother had central visual loss and very small shiny whitish dots in the mid-periphery, with a subnormal scotopic ffERG and flat photopic ffERG. The great aunt primarily had macular atrophy that was more compatible with CACD (Figure 14). Finally, (family 5d) we present a 45-year-old man (F5d-IV-1) with ECA, who reported night blindness and had an abolished scotopic ffERG with a subnormal photopic ffERG.

**Figure 15:**
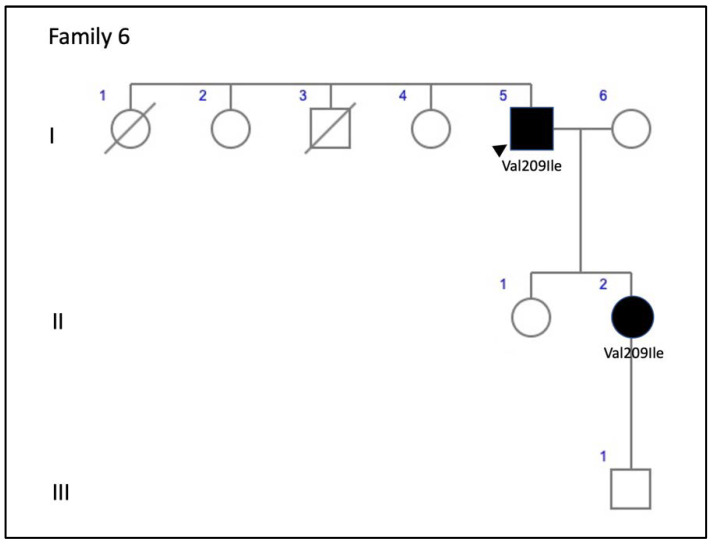
The family 6 pedigree.

Family 6 included a 56-year-old man (F6-I-5) with a mild central vision disturbance and clinical features compatible with AVMD at his first visit, who presented an area of macular atrophy and decreased BCVA after 14 years (Figure 3D–F). F6-II-2 came to the clinic several years later, at the age of 51, presenting with fine parafoveal yellowish deposits in her LE and hyper-AF that caused a small disruption of the RPE line on the SD-OCT (Figure 15).

**Figure 16:**
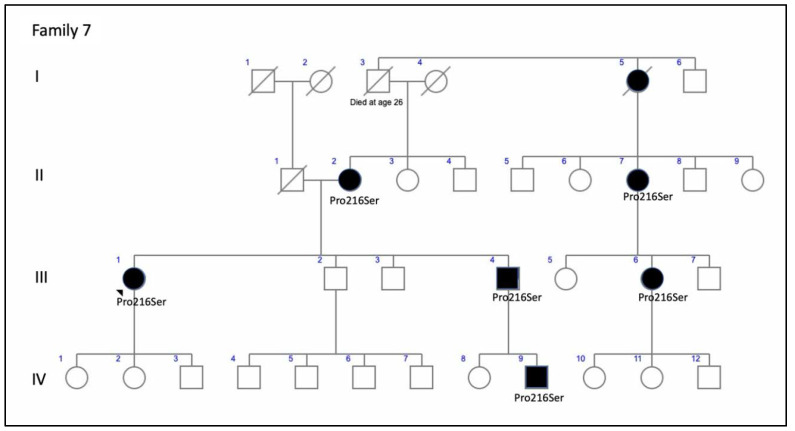
The family 7 pedigree shows a clear autosomal-dominant family tree.

**Figure 17:**
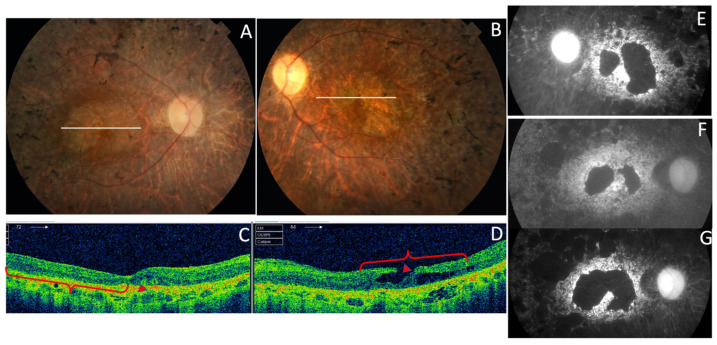
The RP phenotype in the p.Pro216Ser *PRPH2* gene mutation from F7-III-1. (**A,B**) The ocular fundus had pigmented spiculae in the periphery; the SD-OCT images (**C,D**) and autofluorescent images show atrophic macular areas of the LE (**E**) and RE (**F**) at the age of 48 years that increased 19 years later in the RE (**G**), the photopic ffERG was unrecordable at this moment.

F7-III-1, a member of a large family with ADRP, was first examined by us at age 48 years (Figure 16). She presented with pigmented spicules in the retinal periphery and concentric VF retraction (Figure 17). The scotopic and photopic ffERG were abolished (Figure 7).

**Figure 18:**
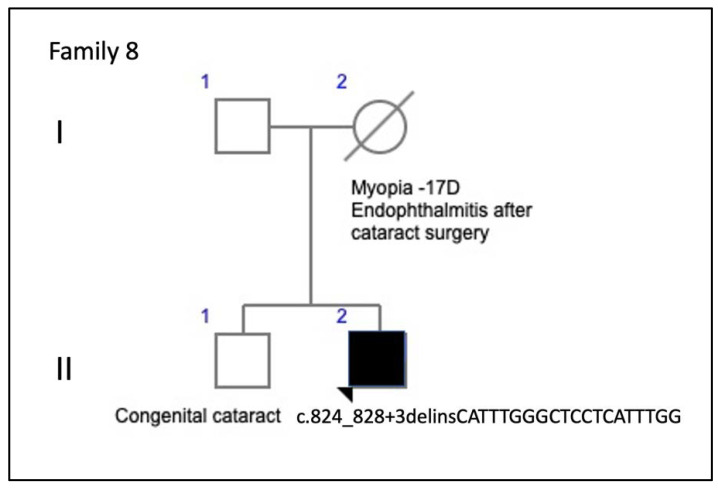
The family 8 pedigree of an apparently isolated case, although the deceased mother had an ocular pathology.

**Figure 19:**
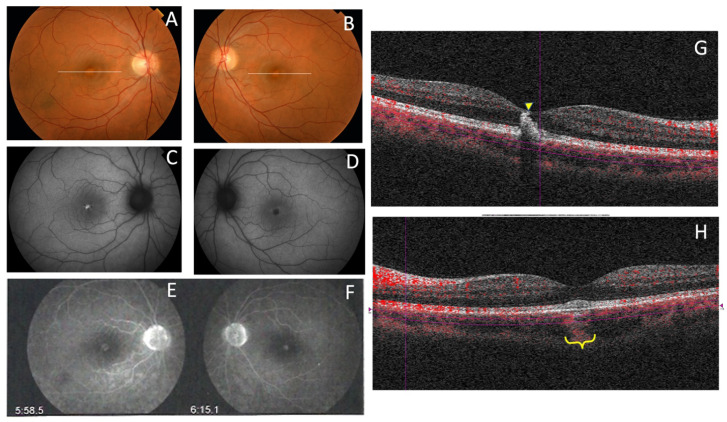
The clinical appearance of the case from F8-II-2 with the new mutation, c.824_828+3delinsCATTTGGGCTCCTCATTTGG. The fundus appearance of AVMD shows (**A**,**B**) yellow foveal deposits, which is (**C**) hyperautofluorescent in the RE and (**D**) hypofluorescent in the LE; (**E,F**) FA shows hyperfluorescence of the lesion in both eyes and (**G,H**) SD-OCT shows hyper-reflective subfoveal deposits bilaterally that (**G**) extend to the inner retina of the RE (yellow arrow) and (**H**) cause choroidal hyper-reflectivity at the foveal region on the LE (yellow brace).

Family 8 included the isolated case of a 34-year-old man (Figure 18), who reported metamorphopsia and central visual loss. He had no family history of ocular disease (Figure 19). The ocular fundus showed a subfoveal yellowish deposit that suggested AVMD (Figure 11). The VF and ffERG were normal.

## 4. Discussion

The association between mutations in the *PRPH2* gene and IRDs was reported first in 1993 [18,19,20]. Currently, to the best of our knowledge, more than 175 pathogenic variants have been described, including missense and nonsense mutations, small in-frame deletions and insertions, small indels, two gross deletions, one gross insertion, and one complex rearrangement, usually heterozygous. They all were associated with retinal phenotypes that could be grouped into three broad categories: ADRP, progressive macular atrophy that sometimes spreads to the mid-periphery, and pattern dystrophies [4].

The relationships between the clinical features and genetic variants are still unclear because the same genetic variant can affect rods and cones differently [21]. In addition, the incomplete penetrance described in several unaffected carriers is not explained by the mutational position in the protein sequence or the mutational type, and the interpretation of genotype–phenotype relationships in animal models is complex [22]. Therefore, without consistent genotype–phenotype correlations, the accepted view is that a single mutation in *PRPH2* may cause a spectrum of phenotypes [21]. Most of these mutations are in the cysteine-rich large intradiscal loop (ID2) (Figure 1), a domain needed to associate peripherin with itself to form homo-oligomers or with its homolog, the ROM1 protein, to form hetero-tetramers and hetero-octamers [8]. We found that most of our patients had central involvement and most mutations were in the large intradiscal loop ID2. We report three missense mutations (p.Gly167Ser, p.Val209Ile, and p.Arg195Leu), a nonsense mutation (p.Arg46Ter), one in-frame deletion (p.Lys154del), and one complex mutation (c.824_828+3delinsCATTTGGGCTCCTCATTTGG). The missense and in-frame mutations were in the intradiscal domains of the PRPH2 protein; in this sense, our hypothesis is that the variant alleles (167Ser, 209Ile or 195Leu, 154del) may alter protein function. To the best of our knowledge, this is the first time that the c.824_828+3delinsCATTTGGGCTCCTCATTTGG genetic variant in heterozygosity was associated with IRDs. In fact, one must be cautious interpreting truncating or splicing variants without a functional assay or familial segregation of the variant and disease. Unfortunately, a segregation analysis in this case was not possible since it was an apparently isolated case and other family members were not available. Nevertheless, this mutation causes a frameshift mutation affecting two residues at the 3’ end of the second exon of the PRPH2 gene, but it also changes the first three bases of the canonical donor splice site of the second intron. This type of variants affecting +1 or 2 splice sites are often assumed to disrupt gene function. Nevertheless, to classify such variants as pathogenic, one must ensure that null variants of these genes are a known mechanism of pathogenicity. We have checked that eleven out of twelve pathogenic or likely pathogenic mutations listed in the ClinVar database downstream from the amino acid residue 275 are null mutations. Moreover, the pathogenic mutation c.828+3A>T (VCV000098713) affects the same donor splice site and other two pathogenic mutations involve the −1 and −4 positions at the 3′ end of the same intron and finally, alternative splicing has not been described in the PRPH2 gene. In summary, in our opinion, the variant c.824_828+3delinsCATTTGGGCTCCTCATTTGG meets the criteria to be considered as a likely pathogenic mutation following the ACMG Standards and Guidelines for the interpretation of sequence variants [23].

To explain the pathogenicity of different genetic variants, it has been hypothesized that mutations induce protein retention in the rod inner segments resulting in a haploinsufficiency/loss-of-function phenotype affecting rods more than cones. Nevertheless, recently, it was reported that normal PRPH2 oligomerization is not required for disc enclosure [24] and that subtle changes can lead to mutant proteins that are sufficiently stable to exert gain-of-function defects in the rods and cones [25]. Moreover, haploinsufficiency may also cause a relatively equal rod and cone cell loss [8]. In addition, some mutations probably induce a dominant negative effect on the rod outer segment structure, resulting in the formation of a dysfunctional PRPH2 tetramer. Finally, other mutations cause a shorter C-terminal domain that may lead to an absent location signal. This highlights the difficulty in targeting PRPH2-associated gain-of-function disease and suggests that the elimination of the mutant protein is a prerequisite for any curative therapeutic strategy and encourages us to continue investigating the basis of *PRPH2* gene-related diseases.

Other factors, such as genetic background, modifying genes, and/or environmental factors, obviously affect phenotypes and outcomes [26]. Modifying genes may include the *ROM1* gene, although it has been reported that variations in this gene do not constitute an important modulating factor [27]; the *RPE65* gene, through modulation of rhodopsin regeneration kinetics and light-damage susceptibility [28]; or the *ABCA4* gene [29]. PRPH2 and ROM1 protein assembly is necessary for proper formation of photoreceptor outer segment discs [5,30], whereas *ROM1* and *ABCA4* genes may alter progression of the related disease with faster progression of visual loss [29]. Concerning this, we report families 2 and 3 with mutations in both the *ABCA4* and the *PRPH2* genes. Interestingly, both showed apparent incomplete penetrance, something that although rare, has been reported previously for *PRPH2* gene mutations [8], and the dominant inheritance pattern was not clearly established before doing the genetic diagnosis of the family. These supposedly healthy family members could not be checked at the genetic level, and they may have had asymptomatic retinal conditions. However, finding multilocus mutations is relevant as the patient from family 2 could have a blended phenotype. Unfortunately, his parents passed away and we could not prove that the mutations in the *ABCA4* gene were in two different alleles. We assume that its modifying effect is more than probable in both families 2 and 3. The phenotype found in family 1 with the p.Arg46Ter *PRPH2* gene mutation had been previously described (AVMD), and the patients had mild visual impairment [13]. However, retinal degeneration became more severe in family 2 when the same mutation was associated with two *ABCA4* gene mutations (p.Leu2027Phe and p.Gly1977Ser). Family 3 had the p.Lys154del *PRPH2* gene mutation, previously described to cause ADRP [14], but our patients’ fundus more resembled Stargardt’s disease, probably due to the effect of the p.Arg2030Gln *ABCA4* gene mutation. Interactions of known genes is a probable scenario for blended phenotypes, however there is a possibility for DNA variations in unknown genes to contribute a completely new phenotype. The targeted NGS testing is unable to exclude or reduce the possibility of unknown gene contributions. Thus, in our opinion it would be advisable to use retinal gene-targeted NGS for genetic diagnosis in any patient, even though the index case belongs to a large family with a known mutation, because this may help identify modifying genes to better explain phenotype–genotype correlations, which must be studied in the future to devise a successful therapy [31]. Nevertheless, targeted NGS gene panel could not be comprehensive enough to identify phenotype modifying variants in unknown genes. Whole exome sequencing (WES) or whole genome sequencing (WGS) are the optimal tools to identify the largest number of these variants. These approaches are able to identify variants potentially related to the primary clinical question. Nonetheless, the interpretation of variants with a subtle effect can be very difficult, especially in single cases or short pedigrees. Moreover, WES and WGS increase the risk of unexpected incidental findings.

Another interesting finding was the new mutation found in family 8 (c.824_828+3delinsCATTTGGGCTCCTCATTTGG), which caused a phenotype of AVMD starting in young adulthood. We also observed that the phenotype was mutation-specific in families 1 and 6 in that patients with the same mutation shared similar clinical pictures and showed the already described phenotype of AVMD [13]. However, the phenotype varied considerably in family 4 with the p.Gly167Ser mutation, which had been reported to cause only PD [32], but we report here multifocal PDsFF, ECA, and ADRP. Differences in age and therefore, disease progression seem to be insufficient to explain the variability of the clinical pictures or outcomes, which had been reported by Boon et al. for other *PRPH2* gene mutations [8].

The very large family with the p.Arg195Leu mutation was very interesting too. This is a founder mutation that probably explains in part the high prevalence of mutations in the *PRPH2* gene found in a Spanish cohort of autosomal-dominant central retinal dystrophies [33]. The mutation was first described to cause CACD [16], but soon after, a more widespread phenotype was described similar to what we found [34]. A limited phenotype variation was observed in this family, as most but not all members presented with ECA and those presenting CACD belonged mostly to the same branch of the family. 

The possible effect of different environmental factors on the phenotype must always be considered [8,35,36,37]. Thus, we advise both caution and not predicting the clinical course and/or the disease severity based solely on the description of a single mutation.

A limitation of this study was the inability to ascertain parental genotypes because it was difficult to determine the de novo or inherited nature of these variants. This was a concern, but parents were not available for the study, although most families had a dominant inheritance pedigree which partly compensated for this limitation of the study. This also prevented from knowing whether the two *ABCA4* gene mutations in the patient from family 2 were in the same allele or not; as a result, we can only hypothesize that this as a blended phenotype. Besides, we did not include the study of copy number variation, which sometimes contributes significantly to pathogenicity in IRDs [38].

In summary, we report a series of patients with diseases associated with *PRPH2* gene mutations. We describe one new mutation (c.824_828+3delinsCATTTGGGCTCCTCATTTGG) associated with the AVMD phenotype. Family 2 presented with the p.Arg46Ter *PRPH2* gene mutation and the p.Leu2027Phe and p.Gly1977Ser *ABCA4* gene mutations jointly, making this a possible blended phenotype. The p.Lys154del *PRPH2* gene mutation, previously described to cause ADRP, caused PDsFF in a current patient, probably due to the effect of the p.Arg2030Gln *ABCA4* gene mutation, which could have worsened the patient’s visual outcome. Finally, we broadened the phenotypic spectrum of two other known *PRPH2* gene mutations (p.Gly167Ser and p.Arg195Leu).

## 5. Conclusions

Considering the current findings, we agree with Jones et al. [39] that families with large phenotypic variations or apparent non-penetrant individuals should raise suspicion for a complex inheritance. Caution should be taken when attributing a single gene disease-causing mutation to a family as a whole, as multiple genes contributing to the phenotype may be discovered using NGS techniques. For this reason, assessing a single mutation should be reconsidered even in families with known mutations. 

## Figures and Tables

**Table 1:** Functional characteristics of patients’ clinical features.

Patient (Gene Variant)	Gender	Age at Onset	Symptoms at Onset	BCVA at First Visit (Age)	BCVA at Last Visit (Age)	Visual Field	ffERG
**F1-II-2** p.Arg46Ter	M	56	Metamorphopsia followed by central vision loss	20/32 RE; 20/25 LE (56)	N/A	Small central scotoma	Normal
**F1-II-3** p.Arg46Ter	Fe	51	Metamorphopsia	20/25 BE (51)	N/A	Normal	Normal
**F1-II-4** p.Arg46Ter	M	46	Metamorphopsia	20/25 BE (46)	N/A	Normal	Normal
**F2-III-6** ***PRPH2***: p.Arg46Ter ***ABCA4***: p.Leu2027Phe/p.Gly1977Ser	M	52	Loss of peripheral visual field	20/50 RE; 20/40 LE (61)	20/63 RE/20/200 LE (76)	Large central scotoma	Scotopic and photopic subnormal 0 dB *b*-wave amplitude = 152 μV RE/182 μV LE; photopic = 22 μV RE/28 μV LE
**F3-V-2** ***PRPH2***: p.Lys154del ***ABCA4***: p.Arg2030Gln	M	37	Metamorphopsia	20/16 RE; 20/20 LE (37)	N/A	Small paracentral scotomas	Scotopic subnormal and photopic preserved 0 dB *b*-wave amplitude = 222 μV RE/219 μV LE
**F4-III-1** p.Gly167Ser	Fe	68	Casual finding after cataract surgery	20/25 BE (68)	20/100 RE; 20/32 LE (78)	Large central scotoma	Scotopic abolished and photopic subnormal Photopic *b*-wave amplitude = 22 μV RE/28 μV LE
**F4-IV-1** p.Gly167Ser	Fe	53	Asymptomatic, casual finding after Dx of her mother	20/20 BE (53)	N/A	Paracentral scotoma	Scotopic and photopic subnormal 0 dB *b*-wave amplitude = 231 μV RE/218 μV LE; photopic = 57 μV RE/61 μV LE
**F4-IV-2** p.Gly167Ser	Fe	51	Asymptomatic, casual finding after Dx of her mother	20/20 BE (51)	N/A	Normal	Normal
**F5a-III-2** p.Arg195Leu	M	53	Loss of central vision	20/32 RE; 20/100 LE (59)	N/A	N/A	Normal
**F5a-III-7** p.Arg195Leu	M	58	Asymptomatic, casual finding after Dx of his brother	20/20 RE; 20/32 LE (58)	N/A	N/A	Normal
**F5a-III-8** p.Arg195Leu	M	59	Asymptomatic, casual finding after Dx of his brother	20/125 RE; 20/100 LE (59)	N/A	N/A	Normal
**F5a-III-10** p.Arg195Leu	M	42	Loss of central vision	20/63 RE; 20/25 LE (55)	20/400 RE; 20/50 LE (61)	Central scotoma	Normal
**F5b-III-4** p.Arg195Leu	Fe	26	Loss of central vision	20/400 BE (89)	N/A	Large central scotoma	Scotopic subnormal and photopic abolished 0 dB *b*-wave amplitude = 87 μV RE/113 μV LE
**F5b-IV-1** p.Arg195Leu	M	39	Loss of central vision	20/25 RE; 20/32 LE (42)	N/A	Central scotoma	Scotopic and photopic subnormal 0 dB *b*-wave amplitude = 228 μV RE/169 μV LE; photopic = 27 μV RE/28 μV LE
**F5b-IV-2** p.Arg195Leu	Fe	31	Asymptomatic, casual finding after Dx of his cousin	20/20 BE (31)	N/A	Small central scotoma	Scotopic and photopic subnormal 0 dB *b*-wave amplitude = 256 μV RE/253 μV LE; photopic = 25 μV RE/27 μV LE
**F5b-IV-4** p.Arg195Leu	M	37	Loss of central vision	20/40 RE; 20/25 LE (37)	20/63 RE/20/25 LE (50)	Large central scotoma	Scotopic subnormal and photopic abolished 0 dB *b*-wave amplitude = 174 μV RE/216 μV LE
**F5c-IV-4** p.Arg195Leu	Fe	25	Loss of central vision	20/800 RE/CF LE (77)	N/A	Central scotoma	Scotopic subnormal and photopic abolished 0 dB *b*-wave amplitude = 231 μV RE/218 μV LE
**F5c-V-2** p.Arg195Leu	Fe	35	Loss of central vision	20/200 RE; 20/125 LE (41)	N/A	Central scotoma	Scotopic subnormal and photopic abolished 0 dB *b*-wave amplitude = 251 μV RE/259 μV LE
**F5c-VI-2** p.Arg195Leu	Fe	18	Loss of central vision	20/50 RE; 20/32 LE (18)	N/A	central scotoma	Scotopic subnormal and photopic abolished 0 dB *b*-wave amplitude = 258 μV RE/299 μV LE;
**F5d-VI-1** p.Arg195Leu	M	20	Loss of central vision and night blindness	20/32 BE (25)	20/100 BE (45)	Large central scotoma	Scotopic abolished and photopic subnormal Photopic *b*-wave amplitude = 35 μV RE/38 μV LE
**F6-I-5** p.Val209Ile	M	56	Metamorphopsia followed by centralvision loss	20/32 RE; 20/25 LE (62)	20/400 BE (76)	First normal, then central scotoma	Normal
**6-II-2** p.Val209Ile	Fe	51	Asymptomatic, casual finding	20/20 BE (50)	20/20 BE (54)	Normal	Normal
**F7-III-1** p.Pro216Ser	Fe	18	Tunnel vision and night blindness	20/20 RE; 20/32 LE (48)	20/25 RE; 20/200 LE (67)	Concentric retraction of visual field	Scotopic and photopic abolished
**F8-II-2** c.824_828+3delinsCATTTGGGCTCCTCATTTGG	M	34	Metamorphopsia	20/25 RE; 20/40 LE (34)	N/A	Normal	Normal

BCVA, best-corrected visual acuity; ffERG, full-field electroretinogram; F, family; C, case; Fe, female; M, male; RE, right eye; LE, left eye; BE, both eyes; *ABCA4*, ATP binding Cassette Subfamily A member 4 gene; *PRPH2*, Peripherin-2 gene; N/A, not available; Dx, diagnosis.

**Table 2:** Image pictures, description of structural changes, and clinical diagnosis.

Patient (Gene Variant)	Fundus at Central Retina	Fundus Rat Peripheral Retina	Autofluorescence	OCT	Clinical Diagnosis
**F1-II-2** p.Arg46Ter	Subfoveal yellowish deposit	Normal	Hyper-AF of the subfoveal deposit	Hyper-reflective deposit above RPE in the foveal/perifoveal regions	AVMD
**F1-II-3** p.Arg46Ter	Subfoveal yellowish deposit	Normal	Hyper-AF of the subfoveal deposit	Hyper-reflective deposit above the RPE in the foveal/perifoveal regions	AVMD
**F1-II-4** p.Arg46Ter	Subfoveal yellowish deposit	Normal	Hyper-AF of the subfoveal deposit	Hyper-reflective deposit above the RPE in the foveal/perifoveal regions	AVMD
**F2-III-6** **PRPH2**: p.Arg46Ter **ABCA4**: p.Leu2027Phe/p.Gly1977Ser	Large confluent areas of CR atrophy, with preserved foveal area	Plaques of CR atrophy	Hypo-AF due to plaques atrophy in macula and periphery of the retina	Outer retinal atrophy of the macular region and choroidal hyper-reflectivity by window defect at the posterior pole in BE. CRT = 128 μm RE/141 μm LE	Extensive CR atrophy
**F3-V-2** **PRPH2:** p.Lys154del **ABCA4:** p.Arg2030Gln	Yellow triradiate flecks in the posterior pole	Yellow and gray triradiate flecks in mid-periphery	Some flecks were hyper-AF and others were hypo-AF	Irregular aspect or disruption of the EZ and IZ limited to the foveal region. CRT = 286 μm RE/287 μm LE	Multifocal PD simulating Fundus Flavimaculatus
**F4-III-1** p.Gly167Ser	Large confluent areas of CR atrophy, with preserved foveal area	Whitish dots in mid-periphery	Confluent hypo-AF areas involving the optic disc and extending beyond the vascular arcades with speckled points of hypo-AF in the posterior pole and mid-periphery	Areas of outer retinal atrophy, EZ and IZ disrupted at the posterior pole in BE. Hyporeflective cysts at the inner nuclear layers and epiretinal membrane in the LE. CRT = 253 μm RE/252 μm LE	ADRP
**F4-IV-1** p.Gly167Ser	Whitish stippling all over the posterior pole	Whitish dots in mid-periphery and small areas of atrophy in far-periphery	Small plaque area of hypo-AF at the fovea with scarce speckled points of hypo-AF and hyper-AF in the posterior pole and mid-periphery	EZ and IZ disrupted at the perifoveal level in BE. CRT = 270 μm RE/272 μm LE	Extensive CR atrophy
**F4-IV-2** p.Gly167Ser	Yellow triradiate flecks in the posterior pole	Yellow triradiate flecks in mid-periphery	Macular hypo-AF with speckled hyper-AF and hypo-AF at the posterior pole and mid-periphery	Irregular aspect or disruption of the EZ and IZ limited to the foveal region. CRT = 264 μm RE/266 μm LE	Multifocal PD simulating Fundus Flavimaculatus
**F5a-III-2** p.Arg195Leu	Atrophy of both maculae with preserved fovea	Normal	Hypo-AF at the atrophic macular area and speckled hypo-AF within the vascular arcades	Outer retinal atrophy of the macular region and choroidal hyper-reflectivity by window defect	CACD
**F5a-III-7** p.Arg195Leu	Whitish stippling only in the macular area	Normal	Hypo-AF at the atrophic macular area	Irregular aspect or disruption of the EZ and IZ at the macular region	CACD
**F5a-III-8** p.Arg195Leu	Atrophy of both maculae	Normal	Hypo-AF at the atrophic macular area	Outer retinal atrophy of the macular region and choroidal hyper-reflectivity by window defect	CACD
**F5a-III-10** p.Arg195Leu	Large areas of CR atrophy and whitish stippling within the vascular arcades	Normal	Hypo-AF at the atrophic macular area and speckled hypo-AF within the vascular arcades	Outer retinal atrophy of the macular region and choroidal hyper-reflectivity by window defect	CACD
**F5b-III-4** p.Arg195Leu	Large confluent areas of CR atrophy in the posterior pole and mid-periphery	Extensive CR atrophy of mid-periphery	Confluent hypo-AF areas involving the optic disc and extending beyond the vascular arcades with speckled points of hypo-AF in the posterior pole and mid-periphery	Outer retinal atrophy of the macular region and choroidal hyper-reflectivity by window defect at the posterior pole in BE	Extensive CR atrophy
**F5b-IV-1** p.Arg195Leu	Whitish stippling all over the posterior pole of BE with macular atrophy (>LE)	Whitish dots in mid-periphery	Small plaque areas of hypo-AF with scarce speckled points of hypo-AF in the posterior pole and mid-periphery	Outer retinal atrophy of the macular region and choroidal hyper-reflectivity by window defect, EZ and IZ disrupted at the posterior pole in BE. CRT = 174 μm RE/184 μm LE	Extensive CR atrophy
**F5b-IV-2** p.Arg195Leu	Atrophy of both maculae with preserved fovea	Normal	Speckled points of hypo-AF in the posterior pole	Retinal thinning of the macular region. CRT = 205 μm RE/213 μm LE	CACD
**F5b-IV-4** p.Arg195Leu	Large confluent areas of CR atrophy, with preserved foveal area at the beginning	Plaques of CR atrophy in mid-periphery	Confluent hypo-AF areas involving the optic disc and extending beyond the vascular arcades with speckled points of hypo-AF in the posterior pole and mid-periphery	Outer retinal atrophy of the macular region and choroidal hyper-reflectivity by window defect, EZ and IZ disrupted at the posterior pole in BE. CRT = 220 μm RE/214 μm LE	Extensive CR atrophy
**F5c-IV-4** p.Arg195Leu	Atrophy of both maculae	Small areas of CR atrophy in the RE	Small plaque areas of hypo-AF in the posterior pole BE and in the mid-periphery RE	Outer retinal atrophy of the macular region and choroidal hyper-reflectivity by window defect. CRT = 179 μm RE/191 μm LE	CACD
**F5c-V-2** p.Arg195Leu	Very small, whitish dots all over the posterior pole	Very small, shining, whitish dots in mid-periphery	Speckled points of hypo-AF in the posterior pole and mid-periphery	Outer retinal atrophy of the macular region and choroidal hyper-reflectivity by window defect, EZ and IZ disrupted at the posterior pole in BE	Extensive CR atrophy
**F5c-VI-2** p.Arg195Leu	Very small, whitish dots all over the posterior pole	Very small, shining, whitish dots in mid-periphery	Speckled points of hypo-AF in the posterior pole and mid-periphery	Retinal thinning of the macular region	Extensive CR atrophy
**F5d-VI-1** p.Arg195Leu	Large confluent areas of CR atrophy, with preserved foveal area at the beginning	Small areas of CR atrophy in BE	Large plaque areas of hypo-AF in the posterior pole and small plaque areas in mid-periphery	Outer retinal atrophy of the macular region and choroidal hyper-reflectivity by window defect at the posterior pole in BE	ADRP
**F6-I-5** p.Val209Ile	Subfoveal yellowish deposit that evolved to macular atrophy	Normal	Hyper-AF first, followed by macular hypo-AF	Hyper-reflective deposit above the RPE in the foveal region followed by outer retinal atrophy of the macular region. CRT = 179 μm RE/164 μm LE	AVMD
**F6-II-2** p.Val209Ile	Drusen-like deposits nasal to fovea in the LE	Normal	Juxtafoveal hypo-AF spots	Small scarce hyper-reflective deposit above the RPE in the perifoveal region. CRT = 226 μm RE/226 μm LE	AVMD
**F7-III-1** p.Pro216Ser	CR atrophy (>LE)	CR atrophy and pigmentation in spicules	Hypo-AF in macula and periphery of the retina	Outer retinal atrophy of the macular region and choroidal hyper-reflectivity by window defect at the posterior pole in BE. Hyporeflective cysts at the inner nuclear layer and lamellar macular hole in the LE. CRT = 188 μm RE/258 μm LE	ADRP
**F8-II-2** c.824_828+3delinsCATTTGGGCTCCTCATTTGG	Subfoveal yellowish deposit	Normal	Hyper-AF deposit in RE and hypo-AF in LE	Hyper-reflective deposit above the RPE in the foveal/perifoveal regions	AVMD

OCT, optical coherence tomography; F, family; C, case; CR, chorioretinal; LE, left eye; RE, right eye; BE, both eyes; AF, autofluorescence; EZ, ellipsoid zone; IZ, interdigitation zone; CRT, central retinal thickness; RPE, retinal pigment epithelium; ADRP, autosomal-dominant retinitis pigmentosa; AVMD, adult-onset vitelliform macular dystrophy; CACD, central areolar choroidal dystrophy; PD, pattern dystrophy.

**Table 3:** Genotype and phenotype data.

Family	*PRPH2* Gene Mutations	*PRPH2* Gene Mutation Type	Location of Mutation in the Prph2 Protein Domain	Global Allele Frequency	Accession Number	Phenotypes of our Patients	All Phenotypes Described
**1** (3 patients)	c.136C>T, p.Arg46Ter	Nonsense	ID1	1.59115 × 10^−5^	rs61755771	**AVMD**: F1-II-2, F1-II-3, and F1-II-4	**AVMD** [13]
**2** (1 patient)	c.136C>T, p.Arg46Ter ***+ABCA4**:* c.6079C>T, p.Leu2027Phe and c.5929G>A, p.Gly1977Ser	Nonsense	ID1	1.59115 × 10^−5^	rs61751408 rs61750639	**ECA**: F2-III-6	**ECA** [current study] (blended phenotype)
**3** (1 patient)	c.461_463del, p.Lys154del ***+ABCA4**:* c.6089G>A, p.Arg2030Gln	Amino acid deletion	ID2	Unknown	rs61755786 rs61750641	**PDsFF**: F3-V-2	**ADRP** [14] **PDsFF** [current study]
**4** (3 patients)	c.499G>A, p.Gly167Ser	Missense	ID2	1.59280 × 10^−5^	rs527236098	**ADRP**: F4-III-1 **ECA**: F4-IV-1 **PDsFF**: F4-IV-2	**PD** [15] **PDsFF** [current study] **ECA** [current study] **ADRP** [current study]
**5** (12 patients)	c.584G>T, p.Arg195Leu	Missense	ID2	3.98349 × 10^−6^	rs121918567	**CACD**: F5a-III-2, F5a-III-7, F5a-III-8, F5aIII-10, F5b-IV-2, and F5c-IV-4 **ECA**: F5b-III-4, F5b_IV-1, F5b-IV-4, F5c-V-2, and F5c-VI-2 **ADRP**: F5d-VI-1	**CACD** [16] **ECA** [13] **ADRP** [current study]
**6** (2 patients)	c.625G>A, p.Val209Ile	Missense	ID2	1.98877 × 10^−5^	rs753657349	**AVMD**: F6-I-5 and F6-II-2	**AVMD** [13]
**7** (1 patient)	c.646C>T, p.Pro216Ser	del	ID2	Unknown	rs61755805	**ADRP**: F7-III-1	**ADRP** [17]
**8** (1 patient)	c.824_828+3delinsCATTTGGGCTCCTCATTTGG	del/ins	TM4	Not previously described		**AVMD**: F8-II-2	**AVMD** [current study]

ID2, intradiscal domain 2; ID1, intradiscal domain 1; TM4, transmembrane domain 4; AVMD, adult-onset vitelliform macular dystrophy; ECA, extensive chorioretinal atrophy; ADRP, autosomal-dominant retinitis pigmentosa; PD, pattern dystrophy; PDsFF: pattern dystrophy simulating fundus flavimaculatus; CACD, central areolar choroidal dystrophy.
